# The Challenge of AIDS-Related Malignancies in Sub-Saharan Africa

**DOI:** 10.1371/journal.pone.0008621

**Published:** 2010-01-11

**Authors:** Annie J. Sasco, Antoine Jaquet, Emilie Boidin, Didier K. Ekouevi, Fabian Thouillot, Thomas LeMabec, Marie-Anna Forstin, Philippe Renaudier, Paul N'Dom, Denis Malvy, François Dabis

**Affiliations:** 1 INSERM, U 897, Epidemiology for Cancer Prevention, Bordeaux, France; 2 Université Victor Segalen Bordeaux 2, Bordeaux, France; 3 INSERM, U 897, HIV in Africa, Institute of Public Health, Bordeaux, France; 4 Hospices Civils de Lyon, Hemovigilance Unit, Lyon, France; 5 Department of Medical Oncology, Yaounde General Hospital, Yaounde, Cameroon; 6 Hôpital Saint André, Tropical Medicine Unit, Bordeaux, France; University of Cape Town, South Africa

## Abstract

**Background:**

With the lengthening of life expectancy among HIV-positive subjects related to the use of highly active antiretroviral treatments, an increased risk of cancer has been described in industrialized countries. The question is to determine what occurs now and will happen in the future in the low income countries and particularly in sub-Saharan Africa where more than two-thirds of all HIV-positive people live in the world. The objective of our paper is to review the link between HIV and cancer in sub-Saharan Africa, putting it in perspective with what is already known in Western countries.

**Methods and Findings:**

Studies for this review were identified from several bibliographical databases including *Pubmed, Scopus, Cochrane, Pascal, Web of Science* and using keywords “*HIV, neoplasia, epidemiology and Africa*” and related MesH terms. A clear association was found between HIV infection and AIDS-classifying cancers. In case-referent studies, odds ratios (OR) were ranging from 21.9 (95% Confidence Interval (CI) 12.5–38.6) to 47.1 (31.9–69.8) for Kaposi sarcoma and from 5.0 (2.7–9.5) to 12.6 (2.2–54.4) for non Hodgkin lymphoma. The association was less strong for invasive cervical cancer with ORs ranging from 1.1 (0.7–1.2) to 1.6 (1.1–2.3), whereas ORs for squamous intraepithelial lesions were higher, from 4.4 (2.3–8.4) to 17.0 (2.2–134.1). For non AIDS-classifying cancers, squamous cell conjunctival carcinoma of the eye was associated with HIV in many case-referent studies with ORs from 2.6 (1.4–4.9) to 13.0 (4.5–39.4). A record-linkage study conducted in Uganda showed an association between Hodgkin lymphoma and HIV infection with a standardized incidence ratio of 5.7 (1.2–17) although OR in case-referent studies ranged from 1.4 (0.7–2.8) to 1.6 (1.0–2.7). Other cancer sites found positively associated with HIV include lung, liver, anus, penis, vulva, kidney, thyroid and uterus and a decreased risk of female breast cancer. These results so far based on a relatively small number of studies warrant further epidemiological investigations, taking into account other known risk factors for these tumors.

**Conclusion:**

Studies conducted in sub-Saharan Africa show that HIV infection is not only strongly associated with AIDS-classifying cancers but also provided some evidence of association for other neoplasia. African countries need now to implement well designed population-based studies in order to better describe the spectrum of AIDS-associated malignancies and the most effective strategies for their prevention, screening and treatment.

## Introduction

Infection with the human immunodeficiency virus (HIV) entails an increased risk of developing cancer [1]. Cancers such as Kaposi Sarcoma (KS), Non Hodgkin Lymphoma (NHL) and Invasive Cancer of the Cervix (ICC) have been recognized for a long time as associated with HIV infection and have been classified as AIDS-defining diseases. More recently, and concomitantly with the lengthening of life expectancy related to the use of Highly Active Antiretroviral Therapy (HAART), an increased risk of other cancers has been found among HIV-positive subjects; they are classified among the non-AIDS defining diseases. This association was originally described in Western countries, where cancer currently accounts for approximately one-third of the causes of death in the patients infected with HIV [2]. The question is now to determine what happens in the low incomes countries and particularly sub-Saharan Africa where more than two-thirds of all HIV-positive people live and where the HAART roll-out is now well under way [3].

The objective of our paper is to review the evidence on the link between HIV and cancer in sub-Saharan Africa, putting it in perspective with what is already known in Western countries. A better understanding of the link between HIV and cancer is indeed necessary for the improvement of care of HIV-infected subjects as well as may shed understanding on the mechanisms of cancer occurrence in the general population.

## Methods

Studies from Africa were identified from several bibliographical databases (*Medline, Scopus, Cochrane library, Web of Science, Francis and Pascal*), using keywords “*HIV*” (including MesH term “*HIV infections*”), “*neoplasia*” (including MesH term “*neoplasms*”) and “*Africa*” (621 references). We then selected all case-referent and observational cohort studies as well as reviews published between 1992 and April 2008 (139 references). For AIDS-defining and non AIDS-defining cancer trends in sub-Saharan Africa, we included data from population-based cancer registries and of course preferably selected studies linking these registries with AIDS registries.

Similarly, references from Western countries were identified from *Pubmed* using keywords “*HIV*” (including MesH term “*HIV infections*”), “*neoplasia*” (including MesH term “*neoplasms*”) and “*epidemiology*” (2448 references). In order to have an overview, as a point of comparison, of what is currently known about the epidemiological association between cancer and HIV in industrialized countries we focused this part of the review on references published since 2000 essentially from studies that linked data from population-based cancer registries and AIDS registries from Europe and North America and Australia (92 references). International Agency for Research on Cancer (IARC) monographs were also included.

Measures of association between HIV and cancer varied in the published materials, depending on study design. All measures, be they Standardized Incidence Ratio (SIR) or Hazard Ratio (HR) in cohort studies or Odds Ratios (OR) in case-referent studies can be taken as estimates of relative risk (RR) and express by how much the risk of cancer is increased in HIV-positive *versus* HIV negative subjects. We report the original measures and their confidence intervals (CI) as they were published.

## Results

### AIDS-defining cancers

#### Kaposi sarcoma

Since the first report by Hymes *et al*, of atypical occurrence of KS among homosexual men in 1981 [4], KS has become the most frequent neoplasm in AIDS patients. Before the HIV epidemic, this mesenchymal tumor involving blood and lymphatic vessels was very infrequent in Western countries with an incidence rate of 0.1 case per one million person-years (py) in Northern Europe and United States of America (USA). Higher rates were observed in countries around the Mediterranean sea [5] where its classical form [6], occurring mostly in elderly men, was originally described. With the HIV epidemic, KS incidence quickly increased in Western countries involving young, more often homosexual or bisexual than heterosexual men or drug addicts. KS-associated herpes virus, also known as Human Herpes Virus 8 (HHV8) is recognized as the necessary cofactor in the pathogenesis of KS, irrespective of the epidemiological setting [7], [8]. Many studies showed that HAART reduced the KS incidence in Western countries. The cohort study “EuroSIDA” reported a decrease in the incidence rate from 24.7 cases (95% CI 17.2–32.2) per 1000 py in 1994 to 4.7 (95% CI 2.7–6.7) per 1000 py in 1997 and 1.7 (95% CI 0.7–3.4) per 1000 py in recent years among HIV-infected individuals [9].

#### Sub-Saharan Africa

**Figure 1:**
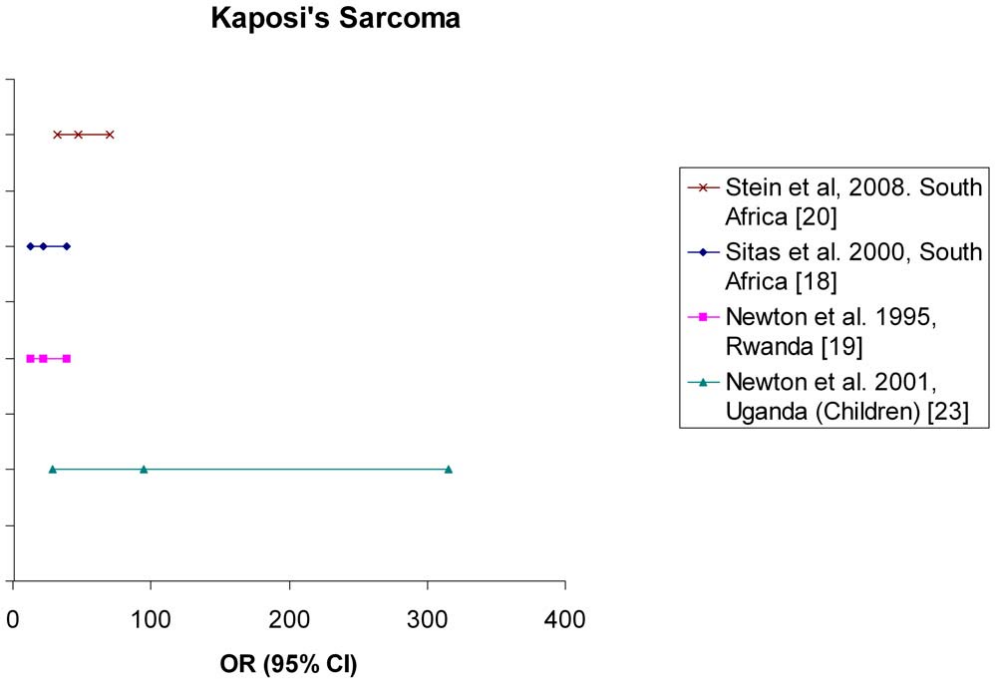
Odds Ratio (OR) and corresponding 95% confidence interval (95% CI) for the association between Kaposi sarcoma and HIV in sub-Saharan Africa.

In sub-Saharan Africa, KS has been much more frequent than in Western countries even before the HIV epidemic, and the African-endemic form has been described since the 1960s. [10]. A role of environmental factors was suggested based on geographical variations [11]. With the AIDS epidemic, the atypical epidemic form of KS has become more frequent in several parts of Africa [12], [13]. It has a commonly aggressive presentation with unusual localisations and frequent visceral organs' involvement. The median survival for childhood AIDS-related KS patients is less than two years [14]. The age at diagnosis is younger than in the general population, affecting now both men and women with two specific peaks: the first in childhood (4–10 years of age) and the second in young adulthood (30–40 years) [15]. The incidence of KS has been steadily climbing in parallel with the AIDS epidemic in sub-Saharan Africa with a 20-fold increase in Uganda and Zimbabwe during the last 15 years making KS the most common malignancy in men and the second most common in women following ICC in these regions [12], [16], [17]. Case referent studies conducted in South Africa and in Rwanda found a clear association between HIV infection and KS with ORs ranging from 21.9 (95% CI 12.5–38.6) to 47.1 (95% CI 31.9–69.8) [18], [19], [20] (Figure 1) whereas a record linkage study in Uganda only found a SIR around 5 to 6 [21]. KS was the second most frequent cancer in children following Burkitt lymphoma in a study conducted in Malawi in 2003 among children (0–15 years) hospitalized for cancer. Among the 707 children enrolled, 61 (9%) had a KS of which 52 were infected by HIV [22]. Newton *et al* found in a case-referent study carried out in Kampala (Uganda) a particularly high risk of KS among HIV-infected compared to HIV-negative children with an OR of 94.9 (95% CI 28.5–315.3) [23] (Figure 1).

#### Non-Hodgkin lymphoma

NHL is the second most common malignant disorder associated with HIV infection worldwide. After the first description in 1982 [24], a study of 90 homosexual men in whom a NHL established the link between AIDS and NHL [25]. High-grade NHL was classified as AIDS-defining in 1985 [26] and large B cell lymphomas were added in 1987 [27]. Studies conducted in Western countries showed that the risk of NHL occurrence among HIV-infected subjects was particularly high compared to HIV-negative subjects with relative risks (RR) of at least 100 [28], [29], [30]. In the context of HIV infection, the classic forms of NHL display a B-phenotype and include Burkitt lymphoma, diffuse large B-cell lymphoma (nodal or primary brain), primary effusion lymphoma and plasmablastic lymphoma of the oral cavity [31]. There is a significant relationship between the subtype of lymphoma and the HIV disease status, with diffuse large B-cell lymphoma occurring in the setting of profound immunodepression (CD4<100.10^6^/L) and Burkitt lymphoma in less immunodeficient patients.

In Western countries, the introduction of HAART induced a significant reduction of the NHL incidence, although less marked than for KS incidence. A first international meta-analysis based on 23 prospective studies compared the incidence of NHL between 1992–96 and 1997–99 [32]. This study confirmed a reduction in the incidence of primary cerebral lymphoma (rate ratio = 0.42 (99% CI 0.24–0.75)) and immunoblastic lymphoma (rate ratio = 0.57 (99% CI 0.39–0.85)). The incidence of Burkitt lymphoma was not reduced (rate ratio = 1.18 (99% CI 0.48–2.88)) [32]. A recent study based on the Swiss HIV Cohort Study evaluated the long-term effect of HAART on NHL incidence. The authors found that the incidence of NHL in 1993–1995 i.e. before HAART introduction was 13.6 cases per 1000 py and declined to 1.8 per 1000 py in 2002–2006. Thus, the use of HAART was associated with a lower risk of NHL among HIV-infected people (HR 0.26 (95% CI 0.20–0.33)) [33]. Moreover, the CD4 cell count was inversely associated with NHL (HR 12.26 (95%CI 8.31–18.07)) in the non-HAART users group whereas only age was significantly associated with the NHL risk in HAART users. The protection HAART confers persists regardless of the HAART regimen [34]. Therefore, the cellular immunosuppression appears to be the main risk factor for NHL, a situation shared by HIV-infected people and transplant recipients [35].

#### Sub-Saharan Africa

**Figure 2:**
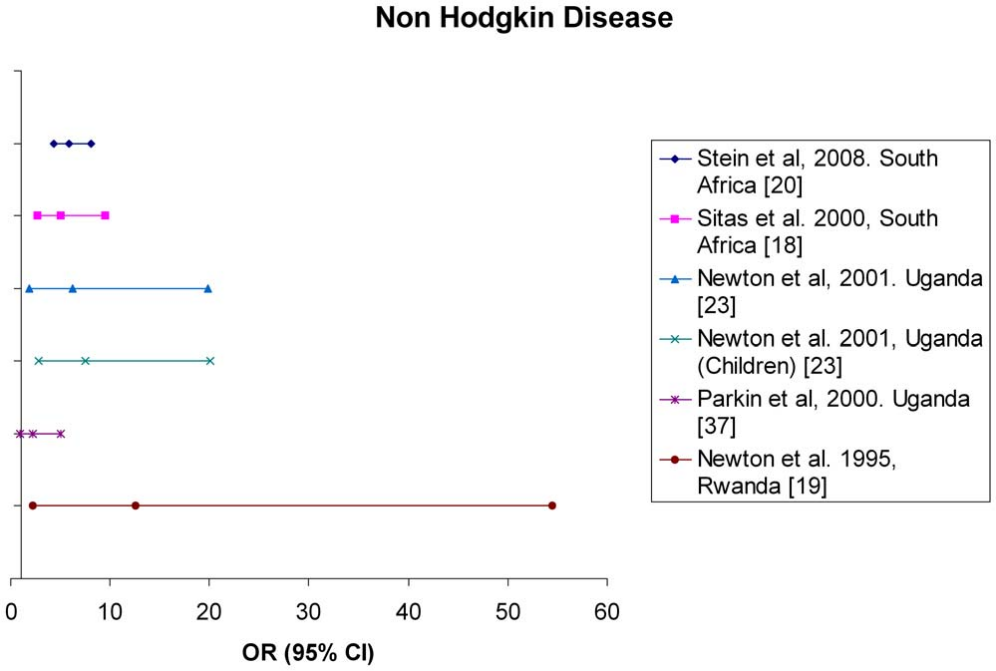
Odds Ratio (OR) and corresponding 95% confidence interval (95% CI) for the association between Non Hodgkin lymphoma and HIV in sub-Saharan Africa.

Several NHL studies have been conducted in sub-Saharan Africa. Data coming from a cancer registry in Uganda showed an overall increase of the NHL annual incidence in the nineties. In Kampala, Uganda, the NHL annual incidence of just more than 3 cases per 100 000 py, stable up to the early nineties, increased to 7.4 per 100 000 py during the 1995–1997 period [16]. Another study examined the frequency and types of AIDS and non-AIDS related malignant lymphoma in Tanzania. This study suggested a general overall trend of increased frequency of malignant lymphoma from the late ‘80s to the mid ‘90s [36]. Several case-referent studies undertaken in South Africa, Uganda and Rwanda from the ‘90’s to the early 2000's found a significant association between NHL and HIV infection with ORs ranging from 5.0 (95% CI 2.7–9.5) to 12.6 (95% CI 2.2–54.4) [18], [19], [20], [23] (Figure 2). A study conducted in Uganda by Mbulaiteye *et al* showed a higher incidence of NHL among HIV-infected people with a Standardized Incidence Ratio (SIR) of 6.7 (95% CI 1.8–17) [21].

In children, malignant lymphoma is mostly represented by endemic Burkitt lymphoma associated with early and persistent Epstein-Barr Virus (EBV) infection. A case-referent study conducted in Uganda by Parkin *et al* involved 132 children with confirmed histological confirmed NHL. Most childhood lymphoma (90%) were EBV-positive Burkitt lymphoma, but no association was found with HIV [37]. In Malawi, another survey conducted in children showed an increase of NHL incidence over time with only one case diagnosed in 1998 *versus* 10 cases in 2002 among the 30 cases of NHL registered during the 6 years of the study. Among the 30 cases, eight (26.7%) had a positive HIV serology. HIV-infected children had a slightly different clinical presentation with lymphadenopathies predominantly located in the head and neck area whereas HIV-negative children had mainly intra-abdominal lymphadenopathies [22].

#### Cervical cancer

According to the World Health Organization (WHO), ICC is the second most common cancer in women worldwide, and is more frequent in low income countries [38]. Annual global estimates around the year 2000 are of approximately 500 000 new cases of, and 230 000 deaths from cervical cancer. Eighty percent of these cases occur in the less industrialized countries [39]. The association between human papilloma virus (HPV), especially 16 and 18 types and ICC is now well established [40]. Women infected with HIV have an increased risk of also being HPV-infected and consequently are at higher risk for cervical cancer [41], [42]. ICC was classified as AIDS-defining in 1993 [43]. In Western countries, women infected with HIV are at a significantly increased risk for in situ and ICCs. A study looking specifically at HPV-associated cancers found for in situ cervical cancer an OR of 4.6 (4.3–5.0) and for invasive an OR of 5.4 (3.9–7.2) [44]. In HIV-negative women, Cervical Intraepithelial Neoplasia (CIN) can regress spontaneously under the influence of the host's immune system. However, a small percentage of cases progress to become ICC. The mechanism by which HIV increases the risk associated with HPV infection may be linked to the immunodeficiency induced by HIV with a poor control of HPV infection [45]. The incidence of this cancer does not seem to have been substantially modified by the introduction of HAART [46], [47], [48].

#### Sub-Saharan Africa

**Figure 3:**
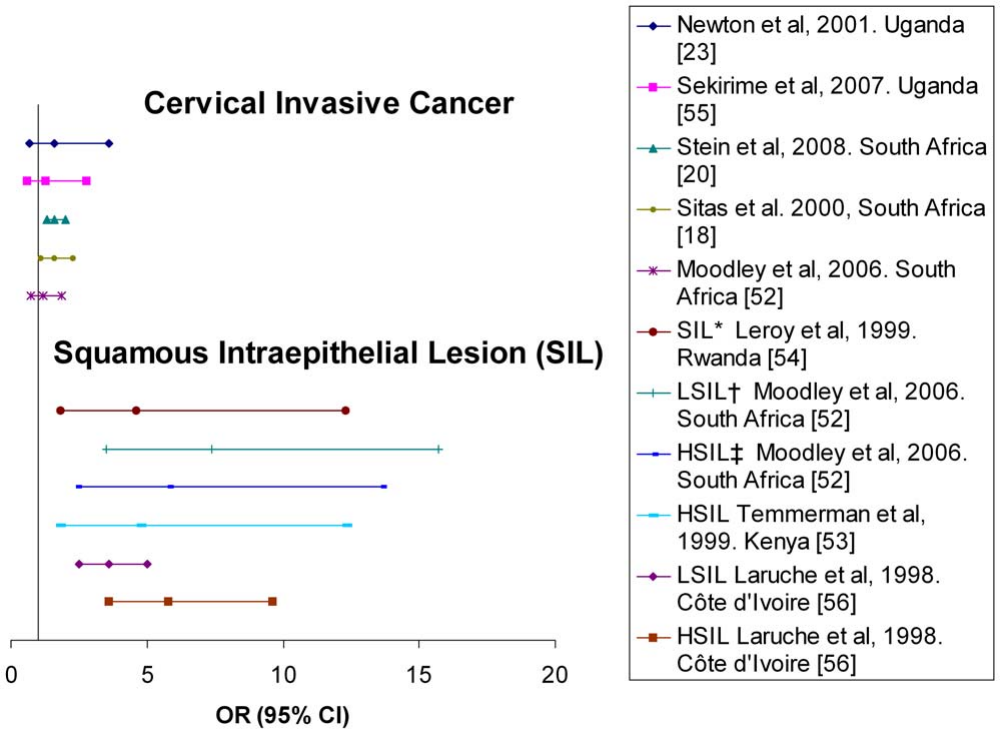
Odds Ratio (OR) and corresponding 95% confidence interval (95% CI) for the association between cervical abnormalities, invasive cervical cancer and HIV in sub-Saharan Africa. * squamous intraepithelial lesions † low-grade SIL ‡ high-grade SIL.

Cervical cancer is much more frequent in sub-Saharan Africa, where it has been highly prevalent even before the HIV epidemic, than in Western countries. Data coming from different cancer registries showed contradictory trends. While the Ugandan tumour registry finds increased cervical cancer rates on the parallel rise of the HIV epidemic [16], the Zimbabwean tumour registry did not identify any increase in the ICC incidence during that same period [49]. Another study conducted in Kenya among 3316 women diagnosed with ICC from 1989 to 1998 highlighted that the three-fold increase in HIV prevalence during this period was not followed by a similar trend concerning ICC [50]. In sub-Saharan Africa, the association between HIV infection and the ICC has been less much strong than the one with KS and NHL. By contrast, several studies conducted in sub-Saharan Africa indicate that among African women, being HIV-infected was associated with a high risk of presenting squamous intraepithelial lesions of the cervix, with ORs ranging from 4.4 (95% CI 2.3–8.4) to 17.0 (95% CI 2.2–134.1) depending on the grade of the lesion and other factors [51], [52], [53], [54] (Figure 3).Yet, many case-referent studies conducted in Uganda [23], [55], Rwanda [19] and Côte d'Ivoire [56] failed to demonstrate any significant association between HIV and ICC, with ORs ranging from 1.1 (95% CI 0.7–1.2) to 1.6 (95% CI 0.7–3.6) (Figure 3).

However, findings from Mbulateye *et al* showed a higher incidence of ICC among HIV-infected patients compared to the general Ugandan population with a SIR of 2.4 (95% CI 1.1–4.4) [21]. Case-referent studies in South Africa also found a significant association with ORs of 1.6 (95% CI 1.1–2.3) and 1.6 (95% CI 1.3–2.0) [18], [20]. An association between HPV and HIV infection has been documented in several studies. According to a recent case-referent study conducted in South Africa, HIV-positive women were nearly five times more likely to have high-risk HPV-infection compared to HIV-negative women (OR 4.6 (95% CI 2.8–7.5))[52]. In another case-referent study conducted in Kenya, HIV-positive women were also more likely to be HPV-infected compared to HIV-negative women (OR 3.1 (95% CI 1.6–5.9)) [57]. In West Africa, a particular attention was given to the association between HIV and cervical abnormalities according to the HIV-1 or HIV-2 status. HIV-1 is the most widespread type worldwide. HIV-2 is less frequent and is essentially found in West Africa where its prevalence represent less than 5% of HIV infection. A study conducted in Senegal found an association between the HIV and HPV infections for the two types of HIV: HIV-1 (OR 2.9 (95% CI 1.7–4.9)) and HIV-2 (OR 1.7 (95% CI 1.0–2.9)) [58]. Concurrently, Hawes *et al* found that, among women infected with high-risk HPVs, HIV-2 infected Senegalese women were at a higher risk to have a high-grade CIN or ICC (OR 6.0 (95% CI 2.1–17.1)) than HIV-1 infected women (OR 2.2 (95% CI 1.0–4.8)) [59]. A previous survey conducted in Côte d'Ivoire by Laruche *et al* found that the prevalence of HIV-2 infection was higher in women suffering from ICC (13%) compared to the referent group without any cervical lesion (2.2%) (P = 0.05). Inversely, the prevalence of HIV-1 in the referent group (17%) did not differ from the HIV-1 prevalence in women suffering from ICC (13%) (P = 0.47) [56]. An association between HIV-2 and squamous intra-epithelial lesions was also found among Senegalese commercial sex workers by Langley *et al* with an OR of 2.9 (95% CI 1.2–7.2) [58]. The pattern of HPV genotypes among HPV-infected women has also been investigated in Africa. A cross-sectional study determined the prevalence of HPV infection, HIV infection, and cervical cytological abnormalities in Ugandan women presenting to a sexually transmitted infection clinic [60]. The authors reported 49 cases of HPV infection among 106 women with cervical swabs adequate for HPV testing (46.2%). Twenty-two cases of HPV infection were identified in the 37 women found to be infected with HIV (59.5%) and 27 in the 69 women not infected with HIV (39.1%). The most commonly detected genotypes of HPV were 52 (14.2%), 16 (7.5%), 58 (7.5%) and 6 (6.6%), the 52, 16 and 58 types being high-cancer risk genotypes and the 6 type being a low risk genotype. Similarly, a study realized in Zambia among 145 HIV infected women of which 93.8% had abnormal Pap smears, found that high-risk genotypes, HPV 52 (37.2%), 58 (24.1%), and 53 (20.7%) were more frequent than HPV 16 (17.2%) and 18 (13.1%) in women with high grade CIN or squamous cell carcinoma [61].

### Non AIDS-defining cancers

#### Squamous cell carcinoma of the conjunctiva

Squamous Cell Carcinoma of the Conjunctiva (SCCC) is a very rare tumor, especially in Western countries where its incidence has been estimated at 0.03 cases per 100 000 py [62]. In the USA, using data from the HIV/AIDS Cancer Match Study, Frisch *et al* found a significant association between this tumor of the conjunctiva and HIV with a RR of 14.6 (95% CI 5.8–30.0) [44]. In a recent publication, using data from the same registry study, Guech-Onguey *et al* confirmed this association (SIR 12.2 (95% CI 6.8–20.2)) in HIV-infected people [63].

#### Sub-Saharan Africa

SCCC has been recognized to be associated with HIV in South Africa, Rwanda, Malawi and Uganda since the early 90's, with ORs ranging from 2.6 (95% CI 1.4–4.9) to 13.1 (95% CI 4.7–37.6) [23], [64], [65], [66]. The other known risk factors for this ocular tumor are intense exposure to solar ultraviolet (UV) radiation and HPV infection [15]. The influence of UV radiation has been established on several occasions. We can cite as example the study published by Newton *et al* in Uganda (2002). In this case-referent study, the risk of SCCC increased with time spent cultivating corresponding to direct sunlight exposure (P_trend_ = 0.05) [67]. Nevertheless the interaction between UV exposure, HPV infection and HIV in the genesis of SCCC is yet to be fully understood.

#### Hodgkin lymphoma

The incidence of HL is clearly increased among HIV-infected patients [68]. Unusual clinical and pathologic characteristics of HL have been described in this setting such as systemic “B” symptoms *i.e.* fever, weight loss or sweats are almost always present. Mixed cellularity and lymphocyte depleted form of classical HL are the predominant pathologic subtypes and bone marrow involvement is found in about half of patients. Previous studies conducted in Western countries showed that HL incidence was 7.8-fold (95% CI 4.4–13) to 11.5-fold (95% CI 8.9–14.6) higher in persons with HIV/AIDS [69], [70], [71] than in the general population. With the use of HAART, the prognosis of HL has improved. In a retrospective cohort study conducted in Germany, Hentrich *et al* found that HIV patients without HAART had a 5.6-fold higher risk (95% CI 2.2–14.3) for 3-year mortality compared to patients under HAART [72]. Another retrospective cohort study conducted in France with a 15-year follow-up found an increase between the 2-year survival rate in 1987–1996 at 45% (95%CI 32.3–57.8) and the 2-year survival rate in 1997–2001 at 62% (95%CI 46.7–77.1) [73]. However, HL incidence in persons with HIV/AIDS had significantly increased since HAART introduction according to the US nationwide study linkage conducted from 1980 to 2002 [69]. This study also displays a complex relation with the CD4 count, in which HL risk reaches a peak in moderately immunosuppressed patients (i.e. 150–250. 10^6^/L) and decreases both in severely immunosuppressed subjects and in patients with a rather normal CD4-cell count.

#### Sub-Saharan Africa

The record-linkage study conducted in Uganda showed an association between HL and HIV/AIDS in Africa with a SIR of 5.7 (95% CI 1.2–17) [21]. In Uganda, Newton *et al* found that two of the four adults with HL (50%) were HIV-seropositive compared to the 24 HIV-seropositive (21%) from the control group (112 patients with cancer not known to be related to an infectious aetiology) [23]. Case-referent studies from South Africa also found an excess of risk of HL among HIV-infected patients with ORs of 1.4 (95% CI 0.7–2.8) and 1.6 (95% CI 1.0–2.7) [18], [20].

#### Lung cancer

HIV-infected persons have an elevated risk for lung cancer, varying from two to seven-fold higher than the general population according to several cohort studies [74], [75], [76], [77], [78]. In 2007, using data from the HIV/AIDS Cancer Match Study, Chaturverdi *et al* confirmed this association with an overall SIR of 3.8 (95% CI 3.6–4.1). This study also highlighted that this association was especially strong among young HIV-infected people aged 15 to 29 years with a SIR of 10.4 (95% CI 3.8–22.7) [79]. A cohort study of intravenous drug abusers conducted by Kirk *et al* has shown the independent role of HIV infection in lung cancer. Indeed, smoking which is the main etiological agent of lung cancer is very common among people with HIV/AIDS and partly explains the increased incidence of lung cancer in this population. After age, sex, smoking status and calendar period adjustment, the relative risk of developing lung cancer was 3.6 (95% CI 1.6–7.9) in HIV-infected *versus* HIV-negative people [80].

#### Sub-Saharan Africa

The record linkage study conducted in Kampala, Uganda, showed a higher incidence of lung cancer in HIV-infected people with a SIR of 5.0 (95% CI 1.0–15.0) [21]. By contrast in a previous case-referent study, Sitas *et al* did not find any association between lung cancer and HIV with an OR of 1.0 (95% CI 0.5–2.3) [18].

#### Hepatocellular carcinoma

The most common risk factors for hepatocellular carcinoma (HCC) are hepatitis B or C virus (HBV, HCV) infection, chronic alcohol intake as well as aflatoxines in the diet in Africa. Although the etiological link between HCC and HBV and HCV is well documented, the influence of HIV infection and immune status on the development of this type of liver cancer is unclear and particularly pertinent for Africa [81]. In the Swiss HIV Cohort study, Clifford *et al.* found a higher rate of liver cancer among HIV-infected people with a SIR of 7 (95% CI 2.2–16.5) [75], similar to an eight-fold risk identified in people with AIDS in the USA [82]. The excess of liver cancer was less clear among HIV-infected people in an early study [83]. This association between HIV and liver cancer found in Western countries may be partly explained by co-infection with HBV and/or HCV among intravenous drug users. Thus, it is difficult to assess the specific role of HIV in the carcinogenesis of liver cancer. Nevertheless, previous publications showed that co-infection with HIV and HCV or HBV led to a higher mortality from liver cancer than HCV or HBV alone [84], [85]. The French GERMIVIC Joint Study Group Network reported an increase in the prevalence of HCC as a cause of death among HIV-infected persons between 1995 and 2001 (respectively 4.7% and 25% of all deaths) [86].

#### Sub-Saharan Africa

In some parts of Africa, especially in West Africa where HBV infection is endemic, liver cancer is highly prevalent. In a publication from the Gambia Liver Cancer Study, HCC was the commonest cancer and 15% to 20% of the total population are chronic HBV carriers [87]. Few studies have evaluated the association between liver cancer and HIV in sub-Saharan Africa. According to the case-referent study conducted by Newton *et al* in Uganda, with a referent group of cancers not related to an infectious etiology, being HIV-infected was only associated with a slight risk of HCC (OR 1.2 (95% CI 0.3–4.2)) [23].

#### Breast cancer

Early studies on breast cancer showed that the incidence was lower in HIV-infected than HIV-negative women [82], [83]. Indeed, in a recent publication from the US HIV/AIDS Cancer Match Registry Study Group, Goedert *et al* found that local stage breast cancer risk among HIV-infected women increased when comparing the 1990–1995 and 1996–2002 cohorts from a SIR = 0.40 (0.22–0.66) to a SIR = 0.61 (0.38–0.93), yet still remaining lower than the risk of the general population. Similarly, the SIRs for regional stage breast cancer went from 0.53 (0.29–0.88) to 0.77 (0.47–1.19) [88].

#### Sub-Saharan Africa

The reduced breast cancer incidence in HIV-infected patients initially described in Western countries has also been found in Africa. Data collected between 1968 and 1996 by the Tanzanian cancer registry showed a significant reduction in the incidence of breast cancer in women as well as men with HIV [89]. One year later, another study emphasized that women below 50 years of age with breast cancer were much less likely to be HIV-infected with an OR of 0.18 (95% CI 0.04–0.76) [90].

#### Other cancers

Anal cancer is closely associated with HIV infection. Bower *et al* showed that the anal cancer incidence rate of 60 cases per 100 000 py was 120-fold higher in HIV-infected persons than in the general population (0.5 cases per 100 000 py) [91]. In another study, the SIR of anal cancer were respectively of 18.3 (95% CI 9.1–32.7) and 19.6 (95% CI 14.2–26.4) in 1980–1989 and 1990–2002 respectively. In the same study, the incidence of penile cancer was higher among HIV-infected men with SIRs of 5.6 (95% CI 1.8–13.1) and 2.2 (95% CI 2.2–20.6) for the same study periods [76].

#### Sub-Saharan Africa

Some HPV-related malignancies such as anogenital cancers (other than cervical cancer) have also be shown to be related to HIV infection with ORs of 4.8 (95% CI 1.9–12.2) and 2.2 (95% CI 1.4–3.3) in the case-referent studies conducted in South Africa [18], [20]. In Uganda, Newton *et al* found an increased risk of penile cancer in HIV-infected men with an OR of 13.0 (95% CI 1.4–122) [23]. Concurrently, the record linkage study from Kampala, Uganda found several cancer sites to be associated with a higher occurrence in HIV-infected populations such as kidney, thyroid and uterus with respective SIRs of 16 (95% CI 1.8–58), 5.7 (95% CI 1.1–16) and 5.5 (95% CI 1.5–14) [21].

Recent findings from a case-referent study conducted by Stein *et al* found that skin squamous cell carcinoma was associated with HIV infection (OR 2.6 (95% CI 1.4–4.9)) [20]. This finding is consistent with other studies from Western countries in HIV-infected patients which found even more increased risks of non melanoma skin cancer with unusual locations [83], [92], [93].

## Discussion

### AIDS-defining cancers

Publications that aimed to assess the association between AIDS-defining cancers and HIV/AIDS in sub-Saharan Africa showed that KS and NHL, known to be directly linked to the severity of immunosupression, were strongly and significantly associated with HIV/AIDS. This association was much weaker for ICC.

For KS, the reduced association observed among African HIV-infected populations compared to the one of industrialized countries might be related to the high background risk of KS among HIV-negative individuals in Africa and the higher seroprevalence of HHV8 reported in sub-Saharan Africa or the influence of other co-factors [94], [95]. The persistence of a high incidence of KS in Africa is in contradiction with its decrease in Western countries, possibly related to the limited use of expensive antiretroviral therapies. Indeed the scale-up of HAART in sub-Saharan countries may be the best way for controlling the disease, immune restoration being the basis of the KS care. However, a small subset of HIV-infected patients with a low CD4+ T-cell count at the initiation of HAART, may develop HIV-KS immune reconstitution inflammatory syndrome (IRIS) within weeks thereafter [96].

A weaker association was found between HIV and NHL in sub-Saharan Africa compared to industrialized countries. This result might be partly explained by the under-ascertainment of NHL in middle and low-income settings, particularly marked for HIV positive subjects. Indeed, from a clinical point of view, as this malignancy requires a costly histological diagnosis it is possible that many patients presenting with polyadenopathies might have been classified as tuberculosis and died without any histological exploration. In addition, NHL cerebral localization has to be differentiated from current infectious opportunistic conditions such as cryptoccocosis (which is quite easy to diagnose), cerebral toxoplasmosis or cytomegalovirus (CMV) infection which remain challenging diagnoses in countries with limited facilities. In this respect, Lucas *et al* conducted a study on a necropsic series of HIV-infected subjects in Côte d'Ivoire and found that 2.8% had histological NHL [97]. A lower susceptibility to NHL among African HIV+ populations has also been hypothesized since in the USA, between 1981 and 1994, the proportion of NHL as an AIDS-defining illness was estimated lower in the black (1%) compared to the white population (3%) [98], although no specific genetic argument definitively supports this hypothesis.

Although there appears to be a clear association between HIV infection and the occurrence of CIN, ICC has been somewhat less strongly associated with HIV in sub-Saharan Africa than in Western countries. Like NHL, this lower association could be due to the competing risk of mortality from other conditions associated with HIV, particularly in settings where HAART is not widely available [52]. A high background risk of ICC among African women may have, to some extent, blurred the impact of HIV on the OR estimates in case-referent studies [99]. The tighter association found between HIV-2 and cervical abnormalities compared to HIV-1 supports the hypothesis that African HIV-1 infected women might die of an opportunistic infection before developing high-grade CIN or ICC, as HIV-2 is known to be less aggressive and having a longer incubation period than HIV-1. Results from several studies conducted in African countries [60], [61], [100], [101] showed that the patterns of HPV genotypes found are quite different from the ones observed in Europe and United States where HPV 16 and 18 (among the high-risk genotypes) predominate in the latter [40]. These findings may have relevant population health implications in sub-Saharan Africa as today most existing HPV vaccines contain only the 16 and 18 carcinogenic genotypes. Therefore, the potential role of HPV vaccination strategies on the African continent still needs to be determined. ICC is the commonest malignancy in women in several African countries, independently from their HIV status. Most women with cervical cancer are presenting with a late-stage disease [102]. These arguments highlight that defining the impact of HIV infection on CIN and ICC has important implications for HIV-infected patients and for population health programs in sub-Saharan Africa since effective and low cost screening tools for cervical screening such as visual inspection of the cervix are already available and need to be implemented [103].

### Non-AIDS defining cancers

The association between HIV infection and HL in Uganda was consistent with previous findings from other studies conducted in Western countries. Inversely, the association between SCCC and HIV infection was primarily and more frequently documented in sub-Saharan Africa rather than Western countries. Even if this difference is partly explained by the higher exposure to solar UV radiations, a well known risk factor of SCCC, the impact of HIV infection on the occurrence of malignancies found in Southern countries may not be directly extrapolated from the Western one. Indeed, confounding factors such as local climatic conditions and particular innate immunity should be sought. Certain non AIDS-defining cancers like skin cancers are also thought to occur with increased frequency or altered course in patients with HIV. This is the case of squamous carcinoma of the skin which shares close patterns with SCCC [83], [92], [93].

There is a real need to describe the patterns of malignancies in African populations as they may not share the same genetic background and as they are not exposed to the same degree to some carcinogenic factors such as solar UV radiation, endemic HBV and EBV infections as well as food contaminations by aflatoxins among others [104]. Recent findings from India by Dhir *et al* emphasized the specificity of the spectrum of HIV/AIDS related cancers in Southern countries. This study conducted in the largest referral cancer centre of Mumbai, India during a four-year period found no case of KS in patients with HIV/AIDS [105]. An important issue in the occurrence of malignancies in HIV-positive individuals is the level of immunodepression as well as its duration. Given the limited availability of HAART in Africa so far, a higher risk of occurrence of KS and to a lesser degree NHL is expected among HIV-positive Africans than HIV-positive in the rest of the world despite the fact that RR of these diseases are lower in the South than in the North. This could also explain the weaker association between HIV and HL. By contrast the role of more frequent co-infections leading to chronic inflammation may be associated with a different tumor profile.

Women now constitute almost half of all AIDS cases. With the increasing use of HAART in sub-Saharan Africa, the overall health and survival of HIV individuals is expected to improve in the coming years. Based on epidemiologic data from Western countries and Africa, HIV infection is not considered as permissive for breast cancer [106]. This may partly reflect the paucity of available data. In addition, breast cancer diagnosis is usually done in women in the second half of their life whereas HIV-infected women are considered until now to have limited life expectancy [90]. Furthermore, screening accessibility in resource-limited countries is still lacking for this condition. Thus, as the HIV population is going to mature, it can be expected that breast cancer in seropositive women will be more often diagnosed.

Results from non AIDS-defining cancers available from the few studies that were conducted so far are based on small-sized populations. This lack of power partly explains the wide confidence intervals associated with estimated SIRs and ORs in these studies. As a number of non AIDS-defining cancers occur with a relatively low incidence, available studies are limited to evaluate the association between cancer and HIV. There is a need for wide and prospective data collection in order to monitor cancer events among HIV-infected persons in low-income settings.

The incidence rate ratio is the most appropriate statistic for making comparisons between populations for which incidence rates are computed. For that purpose the main method to quantify cancer occurrence in an HIV-infected cohort relies on linkage studies between cancer registries and HIV/AIDS databases. Such studies are actually conducted in Western countries [76], [107], where they can rely on population-based cancer registries. Unfortunately, there is a paucity of such cancer registries on the African continent [108]. In this regard, the linkage study conducted in Uganda between the Kampala cancer registry and the AIDS support organization database provided a valuable tool for future investigations because it clearly demonstrates the feasibility of such an approach [21]. Another way to assess cancer occurrence in HIV-infected cohorts is to set up a systematic reporting and registering of cancer on their data collection forms. The International epidemiological Database to Evaluate AIDS (IeDEA) initiative (http://www.iedea-hiv.org) offers an interesting opportunity to set up a standardized case reporting system of malignancies in every region of the world involved in this collaboration (four on the African continent) that will allow valid comparisons between HIV-infected cohorts. By collecting and harmonizing data from many HIV/AIDS cohorts from Western and Southern countries, this initiative will address unique and evolving research questions in HIV/AIDS such as its association with malignancies currently unanswerable by single cohorts.

So far, the epidemiological studies have focused on the strength and precision of the associations between the different malignancies and HIV infection. Not only the new studies will have to consolidate some of these estimates and sometimes reconciliate controversial findings for specific tumors, they will also need to estimate for the first time the general population attributable risk and more importantly perhaps the attributable risk in the exposed (HIV-infected) population that will may deserve focused interventions.

### Conclusion

In Western countries, as HAART has improved the survival of HIV/AIDS patients, the cumulative risk of developing, and dying from, non AIDS-defining cancers is likely to increase. In sub-Saharan Africa, morbidity and mortality are mainly caused by transmissible diseases and complications of HIV-infection are predominantly of infectious origin. With the worldwide scale-up of HAART, chronic conditions such as cancer are likely to represent a growing part in the burden of the HIV-associated morbidity. Some studies in sub-Saharan Africa show that HIV infection is already associated with AIDS-classifying cancers and others provide some evidence of an association between HIV and non-AIDS classifying neoplasia. African researchers need to implement now large and well-designed population-based studies in order to better define the spectrum of HIV-associated malignancies and the most effective strategies for their prevention, screening and treatment.
